# Picky eating in preschool children: Associations with dietary fibre intakes and stool hardness

**DOI:** 10.1016/j.appet.2016.02.021

**Published:** 2016-02-12

**Authors:** Caroline M. Taylor, Kate Northstone, Susan M. Wernimont, Pauline M. Emmett

**Affiliations:** a Centre for Child and Adolescent Health, School of Social and Community Medicine, University of Bristol, Bristol, UK; b School of Social and Community Medicine, University of Bristol, Bristol, UK; c Nestlé Nutrition R&D, King of Prussia, PA, USA

**Keywords:** ALSPAC, Dietary fibre, Constipation, Hard stools, Picky eating, Vegetables

## Abstract

It has been suggested that constipation may be associated with picky eating. Constipation is a common condition in childhood and a low intake of dietary fibre may be a risk factor. Differences in fibre intake between picky and non-picky children and its relation to stool consistency is currently not well-understood. Children enrolled in the Avon Longitudinal Study of Parents and Children identified as picky eaters (PE) were compared with non-picky eaters (NPE): (1) to determine dietary fibre intake at 38 months; (2) to investigate whether any difference in dietary fibre intake was predictive of usual stool hardness at 42 months. PE was identified from questionnaires at 24 and 38 months. Usual stool hardness was identified from a questionnaire at 42 months. Dietary intake was assessed at 38 months with a food frequency questionnaire. Dietary fibre intake was lower in PE than NPE (mean difference −1.4 (95% CI −1.6, −1.2) g/day, p < 0.001). PE was strongly associated with dietary fibre intake (adjusted regression model; unstandardised B −1.44 (95% CI −1.62, −1.24) g/day, p < 0.001). PE had a lower percentage of fibre from vegetables compared with NPE (8.9% vs 15.7%, respectively, p < 0.001). There was an association between PE and usually having hard stools (adjusted multinomial model; OR 1.31, 95% CI 1.07, 1.61; p = 0.010). This was attenuated when dietary fibre was included in the model, suggesting that fibre intake mediated the association (OR 1.16, 95% CI 0.94, 1.43, p = 0.180). Picky eating in 3-year-old children was associated with an increased prevalence of usually having hard stools. This association was mediated by low dietary fibre intake, particularly from vegetables, in PE. For children with PE, dietary advice aimed at increasing fibre intake may help avoid hard stools.

## 1 Introduction

Picking eating is known to result in rejection of specific familiar and unfamiliar foods (Dovey, Staples, Gibson, & Halford, 2008; Taylor, Wernimont, Northstone, & Emmett, 2015), with a reduction in dietary variety and consequently an unhealthy or possibly inadequate diet (Carruth et al., 1998; Jacobi, Agras, Bryson, & Hammer, 2003; Li et al., 2014; Northstone & Emmett, 2013). Its prevalence in developed countries ranges from about 6% to 50% in preschool children (Taylor et al., 2015). The effect of picky eating on dietary fibre intakes, however, is not well documented. Several studies have shown that children who are picky eaters frequently reject, or limit their intake of vegetables (Cardona Cano et al., 2015; Galloway, Fiorito, Lee, & Birch, 2005; Galloway, Lee, & Birch, 2003; Haszard, Skidmore, Williams, & Taylor, 2014; Jacobi et al., 2003; Jones, Steer, Rogers, & Emmett, 2010; Li et al., 2014; Tharner et al., 2014; Xue, Lee, et al., 2015; Xue, Zhao, et al., 2015), which is likely to result in a low intake of dietary fibre. A similar effect would be caused by a low intake of wholegrain products in picky eaters (Cardona Cano et al., 2015; Tharner et al., 2014). There are few studies in which dietary fibre intakes have been measured directly in children with picky eating and compared with intakes in a comparison group: in such studies, dietary fibre intakes have been found to be lower in picky eaters than non-picky eaters but intakes in both groups have generally been found to be below recommended levels (Galloway et al., 2005; Xue, Lee, et al., 2015; Xue, Zhao, et al., 2015). Two further studies have documented low fibre intakes in preschool-age picky eaters but did not include a comparison group (Kwok, Ho, Chow, So, & Leung, 2013; Volger et al., 2013).

It has recently been suggested that constipation may also be associated with picky eating in children. For example, in a study of children attending a Korean paediatric gastroenterology clinic for constipation, being a picky eater was identified as a characteristic by 27% of caregivers compared with only 13% in a control group (Chang et al., 2013). A bidirectional association between picky eating and constipation in preschool children in the Netherlands has also been reported in which there is a 'vicious circle' set up between the two (Tharner et al., 2015). Constipation is a common condition in childhood, affecting up to 30% of school-age children in the UK and accounting for about 3% of general paediatric consultations (Auth, Vora, Farrelly, & Baillie, 2012; Mugie, Di Lorenzo, & Benninga, 2011). In the USA alone, it is estimated to incur annual healthcare costs of US$3.9 billion. Symptoms include reduced frequency of defecation, occurrence of faecal incontinence, stool retention, painful or hard bowel movements, or large diameter stools. Usual treatments include education, toilet training and disimpaction with maintenance therapy and long-term follow-up. For many children, the causes are unknown, but may include genetic predisposition, stool withholding behaviour, cows' milk protein allergy, dietary change or coeliac disease. Fluid intake and physical activity levels may also be important. The primary dietary cause is lack of dietary fibre (Roma, Adamidis, Nikolara, Constantopoulos, & Messaritakis, 1999), and fibre supplements have been shown to be effective in children with chronic constipation (Castillejo, Bullo, Anguera, Escribano, & Salas-Salvado, 2006).

Although constipation seems to be more prevalent in picky eaters, it has not been fully established whether picky eating is associated with lower dietary fibre intakes compared with normal eating and evidence is especially lacking in preschool-age children. It is not known whether low fibre intakes might be caused by rejection of particular fibre-containing foods and/or particular food groups. Finally, it is not known whether dietary fibre is a mediator for possible constipation in this group. The aim of this study was to determine dietary fibre intake, and the relative contribution from food sources, in preschool-age children enrolled in the Avon Longitudinal Study of Parents and Children (ALSPAC) who were identified as picky eaters compared with those who were not picky eaters. A further aim was to investigate the difference in usual stool hardness (as a marker for constipation) between the two groups, and whether dietary fibre intake mediated this difference.

## 2 Materials and methods

### 2.1 The ALSPAC cohort

ALSPAC is a longitudinal population-based study investigating environmental and genetic influences on the health, behaviour and development of children. All pregnant women in the former Avon Health Authority with an expected delivery date between April 1991 and December 1992 were eligible for the study; 14,541 pregnant women were initially enrolled, resulting in a cohort of 14,062 live births with 13,988 alive at 1 year of age (Boyd et al., 2013). The social and demographic characteristics of this cohort at recruitment were similar to those found in UK national census surveys (Fraser et al., 2013). Further details of ALSPAC are available at www.bris.ac.uk/alspac and the study website contains details of all the data that are available through a fully searchable data dictionary (http://www.bris.ac.uk/alspac/researchers/data-access/data-dictionary). Ethics approval for the study was obtained from the ALSPAC Ethics and Law Committee and the Local Research Ethics Committees. The study flowchart is shown in Supplementary Fig. 1.

### 2.2 Defining picky eating in the ALSPAC cohort

The primary caregiver (usually the mother) received a series of postal self-completion questionnaires. The questionnaires are available from the study website (http://www.bristol.ac.uk/alspac/researchers/questionnaires/). A single question on picky eating was asked 24 and 38 months. The question was: 'Does your child have definite likes and dislikes as far as food is concerned?' with possible responses No/Yes, quite choosy/Yes, very choosy. The responses were scored 0, 1 or 2, respectively. A measure of persistence and severity of picky eating was made by combining the scores at 24 and 38 months (combined PE score): 0, score 0 at both time points; 1, score 1 at either or both time points; 2, score 2 once; 3, score 2 at both time points.

### 2.3 Dietary assessment

#### 2.3.1 Food frequency questionnaires

A full food frequency questionnaire (FFQ) was included in the questionnaire at 38 months. The list of foods covered by the FFQ can be found in North and Emmett (2000). Daily intakes of energy, macronutrients and fibre as non-starch polysaccharide (NSP) were estimated (Rogers & Emmett, 1998). NSP broadly includes the cell wall components of plants (including cellulose, hemicelluloses, pectins, gums, mucilages and beta-glucans). It excludes resistant starch or oligosaccharides, which are part of fibre as measured by some other analytical methods, such as that of the AOAC (Department of Health, 1991). Thus fibre intakes measured as NSP are slightly lower than those using AOAC analysis. The FFQ data have been correlated with food record (FR) data collected about 5 months later in the same children (Spearman correlations ranged from 0.12 to 0.33 for nutrients and 0.18 to 0.56 for food groups, all p < 0.001). These correlations were very similar to those found between weighed FRs and a widely used FFQ in a definitive study of dietary assessment methods (0.13 to 0.44) (Bingham et al., 1994). The FR data in ALSPAC have been compared with data from the UK National Diet and Nutrition Surveys (NDNS) of children of a similar age and have been found to be closely related (Emmett, Rogers, Symes, & ALSPAC Study Team, 2002).

#### 2.3.2 Food records

A 10% subsample of the ALSPAC cohort was invited to a research clinic when the children were aged 43 months. Prior to the clinic, parents were mailed a structured diary to record all the foods and drinks that the child consumed over three individual days (one weekend day and two weekdays) in household measures. The FR were checked with the parents in the clinic and then used to calculate daily mean energy, macronutrient and fibre intakes for each child, as described by Emmett et al. (2002). These data were used in this study to confirm data from the FFQ.

#### 2.3.3 Food group sources of dietary *fibre*

Fibre-providing foods were grouped according to type and the weight of the food, the amount of fibre and percentage contribution to total fibre was calculated for each food group.

### 2.4 Stool hardness

Stool hardness was assessed at 30 and 42 months. The caregiver was asked: 'Nowadays how often are his/her stools hard?' with possible responses Usually/Sometimes/Never. This was considered to be equivalent to types 1–3 on the Bristol stool scale (Lewis & Heaton, 1997) and considered to be a marker for constipation as outlined in the UK National Institute for Health and Clinical Excellence guidance on the diagnosis and management of constipation in children (National Institute for Health and Clinical Excellence, 2010). Responses were coded 2, 1, and 0, respectively. Stool hardness at 42 months was used in analyses with dietary variables in order to preserve the temporal sequence of exposure and outcome (stool hardness responses at 30 and 42 months were strongly associated: chi square test p < 0.001, data not shown).

### 2.5 Additional data and confounders

A number of variables were considered as potential confounders: (1) maternal variables (maternal education, pre-pregnancy body mass index, maternal age, maternal fruit and vegetable intake in pregnancy (from an FFQ; aggregate of intake (g/day) of fruit and vegetable items)), Crown–Crisp anxiety subscale (score 0–16) (Crown & Crisp, 1979) and Edinburgh Postnatal Depression Scale (score 0–14) (Cox, Holden, & Sagovsky, 1987) at 21 months postpartum; (2) child variables (birth weight, age of introduction of lumpy foods (Northstone, Emmett, Nethersole, & ALSPAC Study Team, 2001), breast feeding duration).

### 2.6 Statistical analysis

Statistical analysis was carried out with SPSS v21 (IBM Corp.) on singletons only. Analysis of variance was used to investigate any difference in dietary intakes of fibre, and macronutrients and food group sources of fibre, at 38 and 43 months according to picky eating scores at 38 months and the combined PE score. The percentage of children not reaching the proposed UK recommendation for dietary fibre intake for children aged 2–5 years old of 15 g AOAC fibre/day (equivalent to 11 g NSP fibre/day) (Scientific Advisory Committee on Nutrition, 2014) was assessed. Regression modelling was used to evaluate: (1) the association of tertiles of fibre intake at 38 months with stool hardness at 42 months (unadjusted multinomial regression); (2) picky eating at 38 months and combined picky eating score as predictors of dietary fibre intakes at 38 and 43 months (adjusted linear regression: see footnotes in the table for complete details of models); (3) the mediating effect of dietary fibre at 38 months on the association between picky eating at 38 months and stool type at 42 months (unadjusted and adjusted multinomial regression: see footnotes in the table for complete details of models).

## 3 Results

### 3.1 Prevalence of picky eating

The prevalence of high picky eating scores at 24 and 38 months are shown in Table 1, together with the combined score variable for 24 and 38 months taken together. More children were described by parents as 'quite choosy' or 'very choosy' at 38 months than at 24 months. When the two ages were combined 6.4% of children were very choosy at both ages (combined PE score 3) with 39.2% never choosy (combined PE score 0).

### 3.2 Dietary intakes

To assess differences in diet according to picky eating score, FFQ data were available for 9544 children and FR data were available for 815 children. The differences in intakes of energy, macronutrients and fibre by picky eating score at 38 months and by the combined picky eating score are shown in Table 2 for the FFQ and Supplementary Table 1 for the FR. The differences in fibre-contributing food groups by picky eating score are shown in Table 3 for the FFQ and Supplementary Table 2 for the FR. The percentage of children failing to reach the proposed UK recommendation for dietary fibre intake (Scientific Advisory Committee on Nutrition, 2014) by picky eating score at 38 months and for the combined picky eating score are also shown. Even for children without picky eating, just over 75% consumed less than the recommendation, rising to over 85% in picky eating children for the FFQ (Table 1). The percentages were slightly higher in the FR (Supplementary Table 2).

#### 3.2.1 Cross-sectional picky eating score

Based on FFQ data there were differences in fibre, energy and all the macronutrient intakes investigated according to PE score. However, the differences for energy, fat, carbohydrate and free sugars were very small (<6% of the total) and were not replicated in the FR data. There was a difference in protein intakes between the 'very choosy' (score 2) and the 'never choosy' (score 0) groups of 12% by FFQ; this was partially confirmed by the FR with a 7% difference in protein intake. The most substantial difference was in dietary fibre intake, which was ~15% lower in the 'very choosy' children compared with 'never choosy' children by FFQ, and this was confirmed by the FR as ~17% lower. In general, the 'quite choosy' children had intakes which were more similar to the 'never choosy' than the 'very choosy' children.

Analysis of the dietary sources of fibre from the FFQ showed that bread was consistently the main contributor to dietary fibre intake (~19%), followed by vegetables (~16%), cereal (~15%) and fruit (~9%) (Table 3) in the 'never choosy' group; the FR showed similar results (Supplementary Table 2). The deficit in dietary fibre in children who were picky eaters compared with non-picky eaters was largely driven by a reduction in vegetable consumption (Table 3 and Supplementary Table 2). Data from the FFQ showed that 'very choosy' children consumed 52% fewer vegetables (by weight) than those who were 'never choosy' (Table 3), resulting 6.8 g/week less fibre consumed. This finding is supported by data from the FR (48% less weight of vegetables and 4.8 g/week less fibre consumed) (Supplementary Table 2).

Other food groups also contributed to the overall lower dietary fibre intake in the 'very choosy' compared with the 'never choosy' group. Cereal intake was lower in the FFQ (Table 3) by 15%, fruit intake by 14.6%, rice/pasta intake by 17.2%, boiled/mashed potatoes intake by 31.3% and baked beans intake by 28.6%. Conversely, intake of crisps was slightly higher in children who were picky eaters in the FFQ by 7.5%. Only the lower fruit and slightly lower baked bean intakes were supported by the FR data (Supplementary Table 2).

#### 3.2.2 Longitudinal picky eating score

When the children who were 'very choosy' at both ages (score 3) were compared with those who were 'never choosy' (score 0) similar differences to those above were found. The deficit in vegetable and fruit intake in children who were picky at both ages was more pronounced than for children who were only picky at one age (Table 3 and Supplementary Table 2).

### 3.3 Picky eating as a predictor of fibre intake

For both PE definitions, about 2.5% of the variation in fibre intake from the FFQ was explained by minimally adjusted PE score; for the FR data slightly more of the variation was explained by the minimally adjusted score (~5%) (Table 4 and Supplementary Table 3). Each of the models adjusted using different variables from the literature explained more of the variation, with adjustment for the maternal diet in pregnancy and maternal anxiety and depression being the most effective (~11%). The final model adjusting for all the literature variables together explained about 13% of the variation in fibre intake from both the FFQ and FR. In the fully adjusted models there was very little difference between the FFQ and FR in the reduction of fibre consumed by the children with the highest compared with the lowest PE scores (FFQ −1.70 (95% CI −1.96, −1.43); FR −1.88 (95% CI −2.73, −1.04) g/day).

### 3.4 Hard stools in relation to fibre intake and picky eating scores

The mean fibre intake was low in this cohort of children (8.8 ± 2.9 g NSP/day): about 77% of the proposed UK Scientific Advisory Committee on Nutrition (SACN) recommended intake of 15 g AOAC fibre/day (equivalent to 11.3 g NSP fibre/day) for children aged 2–5 years (Scientific Advisory Committee on Nutrition, 2014). Tertiles of fibre intake were associated with stool hardness (Table 5) such that the lowest fibre intake groups were almost twice as likely to 'usually' have hard stools compared with the highest fibre intake group. Children with higher picky eating scores were less likely to never have hard stools than non-picky children (Supplementary Table 4). The adjusted odds of a child with picky eating usually having hard stools was 31% higher than for a non-picker eater (odds ratio 1.31, 95% CI 1.07, 1.61; p = 0.010) (Table 6); however, this association was strongly attenuated after adjustment for fibre intake from the FFQ (odds ratio 1.16, 95% CI 0.94, 1.43; p = 0.180) (unadjusted data shown in Supplementary Table 5). Adjusted and unadjusted data for FR confirmed these results (data not shown). It is likely therefore that dietary fibre intake mediates the relationship between stool hardness and picky eating status.

## 4 Discussion

Preschool children who were considered by their parents to be 'very choosy' about food (defined as picky eaters) consumed less dietary fibre than those who were 'never choosy' (non-picky eaters), but did not have consistently lower dietary energy and macronutrient intakes in either cross-sectional analyses or longitudinal analyses. The overall intake of fibre was low compared with recommendations and there was a high incidence of children usually having hard stools (29%). The children who were picky eaters were 30% more likely to have hard stools than the non-picky eaters and this was explained by their fibre intake. The food group intake most strongly affected by picky eating status was vegetables: picky eaters ate about half the amount eaten by non-picky eaters. A lower fruit intake was also found in the picky eaters but was less marked than for vegetables.

To date, dietary fibre intakes have generally been poorly characterised in children who are picky eaters. Some studies have focussed on older children (Galloway et al., 2005; Xue, Lee, et al., 2015) or have omitted to include a control group of non-picky eaters (Kwok et al., 2013; Volger et al., 2013). Fibre intake in US girls who were identified as picky eaters at 9 years old was significantly lower than in non-picky eaters (11.2 vs 12.7 g/day, respectively), but both groups failed to meet the US recommendation for fibre (Galloway et al., 2005). In China fibre intakes in picky eaters were lower than in non-picky eaters (6.8 vs 7.6 g/day for 3–7-year olds (Xue, Zhao, et al., 2015) and 5.0 vs 6.4 g/day for 7–12-year-olds (Xue, Lee, et al., 2015)). Two further studies quantified fibre intakes in preschool age picky eaters but did not include a reference group: 8.1 g/day in Chinese preschoolers (Kwok et al., 2013) and 7.3 g/day in Chinese/Hong Kong preschoolers (Volger et al., 2013). However, there is consistent evidence that children who are picky eaters reject or limit their intake of vegetables (Cardona Cano et al., 2015; Galloway et al., 2005; Galloway et al., 2003; Haszard et al., 2014; Jacobi et al., 2003; Jones et al., 2010; Li et al., 2014; Tharner et al., 2014; Xue, Zhao, et al., 2015) and have a lower intake of wholegrain products than non-picky eaters (Cardona Cano et al., 2015; Tharner et al., 2014): this is likely to result in a low intake of dietary fibre as vegetables and cereals are the main sources of fibre in children's diets (Gregory, Collins, Davies, Hughes, & Clarke, 1995). To our knowledge, the present study is the first to document sources of fibre intake in picky versus non-picky preschool age children in the UK. In addition, this is first time that the association between picky eating and stool hardness, and the mediating effect of dietary fibre, has been studied, although a bidirectional association between fussy eating and functional constipation in preschool children has been found previously in the Netherlands (Tharner et al., 2015).

The overall dietary intake of energy and macronutrients of these children was similar to that of NDNS, a nationally representative sample of UK children studied cross-sectionally in 1992/3, at about the same time as the present study (Gregory et al., 1995); however, the fibre intake of children in ALSPAC was slightly higher than in NDNS 1992/3 (1.5–4.5 years old; 8.6 vs 6.6 g/day, respectively). A more recent iteration of NDNS in 2008–2012 has shown an increase in UK children's fibre intakes to 8.2 g/day in 1.5–3-year-olds, a value closer to that found in ALSPAC 20 years previously (Bates et al., 2014). Food sources of fibre in this study were in slightly lower proportions to those in the NDNS 2008-2012 in which vegetables provided 15% of dietary fibre, and fruit provided 16%, suggesting a small increase in intakes of vegetables and fruit in recent years. All these groups had very low fibre intakes compared with the proposed UK SACN guideline of about 15 g AOAC fibre/day for children aged 2.0–5.0-years-old (Scientific Advisory Committee on Nutrition, 2014), equivalent to about 11 g NSP fibre/day. In the NDNS 1992/3 (Gregory et al., 1995), fibre intake was positively associated in a dose–response manner with the number of bowel movements per day, and this is similar to our finding in the present study where fibre intake was associated with stool hardness (Table 5). These comparisons suggest that findings from this study may be generalisable in the UK over time and possibly in other countries with a western-style diet.

Although the FFQ data showed some statistically significant differences between the picky and non-picky eaters for intakes of energy, fat and carbohydrate in both cross-sectional and longitudinal analyses, these differences were very small (<6%) and the amounts were not inadequate in the diet (Scientific Advisory Committee on Nutrition, 2011). In addition, these differences were not supported by the FR data. There were slightly larger differences in protein intake from the FFQ that were also present in the FR data; however, protein intakes in all the children were much higher than the UK recommended intakes for children of this age (Department of Health, 1991), so it is unlikely to be a great cause for concern.

Both fruit and vegetable intakes were lower in the picky eaters than the non-picky eaters. The UK recommended intake of fruit and vegetables for health is five portions a day (NHS Choices); based on the relative energy intake of a 3-year-old child compared with an adult this would equate to ~250 g/day for a child (Glynn, Emmett, Rogers, & ALSPAC Study Team, 2005). The mean total weight of fruit and vegetables consumed by non-picky eaters using FR data was 847 g/week (~120 g/day), half of the recommended amount. The picky children were consuming a mean total weight of 519 g/week (~74 g/day), less than one-third of the recommendation (NHS Choices). Increased intakes of fruits and particularly vegetables should be encouraged in all children, and research has shown that even in 'picky' children repeated exposure to vegetables increases intake gradually (Caton et al., 2013; Caton et al., 2014). The UK SACN draft guidelines on carbohydrates and health recommends that fibre intake should be achieved from a variety of foods as it is not known if extracted or isolate dietary fibres would convey the same range of health benefits associated with the consumption of dietary fibre rich foods (Scientific Advisory Committee on Nutrition, 2014). An increased consumption of fruits and vegetables would help towards this goal.

The presence of hard stools is one of the symptoms of constipation described in the UK National Institute for Health and Clinical Excellence (NICE) guidelines on constipation in children (National Institute for Health and Clinical Excellence, 2010). Picky eaters were 30% more likely to have this symptom of constipation than non-picky eaters. In this study we found that fibre intake was associated with the presence of hard stools both in the whole cohort (Table 5) and in the picky eaters (Table 6), suggesting that increasing fibre intake in the whole cohort of children may lead to improved bowel habits.

One of the strengths of this study is its ability to follow a relatively large cohort of free-living children longitudinally so that the presence of childhood problems can be ascertained and the possible consequences followed over time. The study has collected dietary information at intervals during childhood by two different but complementary methods. The FFQ was completed by parents in the whole cohort, but the FR was only obtained from a subsample. The FR has more individual detail than the FFQ and can provide very good estimates of the amounts of foods consumed and their nutrient content (Bingham et al., 1994). However, both methods have the same systematic errors of misreporting of particular foods and memory bias (Livingstone, Robson, & Wallace, 2004). In this case it is likely that the FFQ underestimated fruit intake and overestimated vegetable intake since it had only one question covering fruit but six questions covering vegetables. Despite these differences the FFQ and FR both provided evidence that picky eaters were lower consumers of fruit and vegetables than non-picky eaters. The study was carried out in one geographically defined area of the UK but comparisons with dietary intakes from nationally representative children of a similar age showed similar food and nutrient intakes (Gregory et al., 1995). The longitudinal picky eating score encompassed a relatively long period of time (24–38 months). It was constrained by the need to maintain a temporal sequence of exposure and outcome (stool hardness variable at 42 months) but it did include the age of peak prevalence of picky eating in this cohort (Taylor et al., 2015). There was lower power in the FR data than in the FFQ data as data were collected from only a subsample of the cohort, limiting comparability of data, but the FR is generally regarded as a relatively accurate method of capturing food and nutrient intake and is often used as a comparator for other methods (Emmett, 2009). A recent investigation of the validity of an FFQ compared with an online FR in preschool children indicated that the FFQ tended to overestimate fibre intake by about 13% (Vereecken, Rovner, & Maes, 2010). In the present study, mean fibre intakes measured in the FFQ were slightly higher than in the FR, but only by about 6%. There was evidence for a correlation between the two estimates of fibre intake in children with both measures of diet: Spearman's r 0.33, p < 0.001. Any differences in the measures do not detract from our overall finding that mean fibre intakes were well below proposed recommended intake in the non-picky children, and were even lower in picky eaters. There are some limitations to the study. First, although dietary fibre is not traditionally considered to be a nutrient, it is essential for health and the limitations of a single nutrient approach are still highly relevant. These limitations are the lack of account taken of the interactions and synergistic effects of a range of nutrients, as well as placing undue emphasis on the deficient nutrient without consideration of the context of the whole diet. For example, dietary fibre is often found in association with phytochemicals that may also affect gut health: we were not able to distinguish these complex effects in this observational study. For a complex exposures such as diet, multiple approaches to determine the relationship with disease risk are ideal. Second, picky eating scores were derived from one question asked to parents in two self-completion questionnaires completed when their child was aged 24 and 38 months. This was an unambiguous question about child choosiness and is similar to those used in several recent studies (Goh & Jacob, 2012; Jani Mehta, Mallan, Mihrshahi, Mandalika, & Daniels, 2014; Mascola, Bryson, & Agras, 2010; Orun, Erdil, Cetinkaya, Tufan, & Yalcin, 2012), but did not cover the full range of 'picky eating' traits as defined in some other studies (Taylor et al., 2015). However, the question did not invite the parents to define picky eating for themselves. Third, information on stool hardness was derived from questions completed by untrained parents who might interpret the question in various ways. Finally, the minimally adjusted PE score explained only a small proportion of the variation in fibre intake (about 2.5% (FFQ data) or 5% (FR data)) and the final, fully adjusted models considering all literature variables together explained about 13% (for both FFQ and FR) of the variation in fibre intake; these findings suggest that many other factors may affect the dietary fibre intake of children in this age group, and that there are possible confounders that we have not been able to take into account (for example, antibiotic use, fluid intake, physical activity).

## 5 Conclusion

We found consistent evidence that children who are picky eaters defined by parental questionnaire consume less vegetables and fruit than non-picky children and that this contributes to a lower dietary fibre intake in children who are picky eaters. We have also shown that picky eaters are more likely to usually have hard stools than non-picky eaters and that their fibre intake mediates this association. This research highlights the need to increase the fibre intake of the majority of children, particularly by increasing their vegetable and fruit intakes. Parents of children who are picky eaters would benefit from increased levels of advice and support when trying to achieve this aim. The best advice includes use of a combination of approaches, including repeated offering of vegetables to overcome neophobia, parental example in eating vegetables, and regular family mealtimes with the same meal offered to but not forced on all participants.

## Supplementary Material

Supplementary data related to this article can be found at http://dx.doi.org/10.1016/j.appet.2016.02.021.

Supplementary data

## Figures and Tables

**Table 1:** Picky eating scores for children aged 24 and 38 months in ALSPAC derived from parent-completed questionnaires at each age, and a combined picky eating score derived from both questionnaires taken together.

	Picky eating score			
	0	1	2	3
PE score: 24 months^a^	6039 (59.6%)	3113 (30.7%)	982 (9.7%)	–
PE score: 38 months^a^	4448 (45.2%)	3948 (40.1%)	1448 (14.7%)	–
Combined PE score^b^	3456 (39.2%)	3866 (42.6%)	1074 (11.8%)	585 (6.4%)

Values are n (%).

a PE score: Does your child have definite likes and dislikes as far as food is concerned? 0, no; 1, yes, quite choosy; 2, Yes, very choosy.

b Combined PE score: 0, score 0 at both time points (24 months and 38 months); 1, score 1 at either or both time points; 2, score 2 once; 3, score 2 at both time points. Adapted from Taylor et al. (2015).

**Table 2:** Fibre and macronutrient intakes from FFQ in children in ALSPAC aged 38 months by picky eating score at 38 months and a combined picky eating score for 24 and 38 months.

	Diet at 38 months (FFQ)			

	0^a^	1^b^	2^b^	3
PE score at 38 mo^c^				
n	4307	3837	1400	–
Fibre (g/day)	9.1 (9.0, 9.2)	–0.4 (–0.5, –0.2)***	–1.4 (–1.6, –1.2)***	–
% below UK proposed RDI^e^	77.8	80.7	86.2	–
Energy (kJ/day)	5307 (5267, 5346)	–47 (–116, 23)	–255 (–353, –158)***	–
Carbohydrate (g/day)	167 (166, 168)	–1 (–3, 2)	–6 (–9, –2)***	–
Fat (g/day)	50.1 (49.7, 50.5)	–0.2 (–1.0, 0.5)	–1.8 (–2.8, –0.8)***	–
Protein (g/day)	45.8 (45.5, 46.2)	–1.5 (–2.1, –0.9)***	–5.5 (–6.3, –4.6)***	–
Free sugars (g/day)	48.2 (47.6, 48.8)	0.9 (–0.2, 2.0)	1.7 (0.1, 3.2)*	–
Combined PE score^d^				
n	3455	3766	1040	568
Fibre (g/day)	9.2 (9.1, 9.3)	–1.4 (–0.6, –0.2)***	–1.1 (–1.4, –0.9)***	–1.6 (–2.0, –1.3)***
% below UK proposed RDI^e^	77.4	80.9	85.4	86.4
Energy (kJ/day)	5303 (5259, 5346)	–65 (–147, 16)	–175 (–297, –52)***	–315 (–472, –158)***
Carbohydrate (g/day)	167 (165,168)	–2 (–4, 1)	–5 (–9, –1)*	–8 (–13, –2)***
Fat (g/day)	50.0 (49.6, 50.5)	–0.4 (–1.2, 0.5)	–1.0 (–2.2, 0.3)	–2.2 (–3.8, –0.5)**
Protein (g/day)	45.6 (45.5, 46.2)	–1.4 (–2.1, –0.8)***	–3.7 (–4.7, –2.6)***	–6.7 (–8.0, –5.4)***
Free sugars (g/day)	48.0 (47.3, 48.6)	0.4 (–0.8, 1.7)	1.1 (0.8, 3.0)	1.8 (–0.7, 4.2)

FFQ, food frequency questionnaire; PE, picky eating; RDI, recommended daily intake. Dietary fibre is measured as non-starch polysaccharide (NSP). Values significantly different from 0 category: *p ≤ 0.05, **p ≤ 0.01, ***p ≤ 0.001 (ANOVA with multiple comparisons).

a Values are mean (95% CI); singletons only.

b Values are mean differences (95% CI) from reference category; singletons only.

c PE score: Does your child have definite likes and dislikes as far as food is concerned? 0, no; 1, yes, quite choosy; 2, yes, very choosy.

d Combined PE score: 0, score 0 at both time points (24 months and 38 months); 1, score 1 at either or both time points; 2, score 2 once; 3, score 2 at both time points.

e Proposed UK guideline of 15 g AOAC fibre/day for children ages 2–5 years old (equivalent to 11 g NSP fibre/day) (Scientific Advisory Committee on Nutrition, 2014).

**Table 3:** Main food group sources of dietary fibre (non-starch polysaccharide) (food weight in g/week, g fibre/week and % of total fibre) assessed in children in ALSPAC by parental-completion FFQ at age 38 months by picky eating score at age 38 months and by combined picky eating score for ages 24 and 38 months.

	Diet at 38 months (FFQ)				
	0^a^	1^b^	2^b^	3	P value^c^
**PE score at 38 mo^d^**					
n	4307	3837	1400		
Potatoes					
Chips/roast					
Weight (g/week)	215 (211, 219)	3 (−4, 10)	6 (−5, 16)	−	0.578
Fibre (g/week)	4.3 (4.3, 4.4)	0.1 (−0.1, 0.2)	1.8 (0.0, 0.4)	−	0.124
Fibre (% of total fibre)	7.3 (7.1, 7.4)	0.6 (0.3, 0.9)	1.8 (1.4, 2.2)	−	<0.001
Boiled/mashed					
Weight (g/week)	211 (208, 215)	−13 (−20, −6)	−66 (−75, −56)	−	<0.001
Fibre (g/week)	2.4 (2.4, 2.5)	−0.15 (−0.23, −0.07)	−0.8 (−0.9, −0.6)	−	<0.001
Fibre (% of total fibre)	4.0 (3.9, 4.0)	−0.1 (−0.2, 0.1)	−0.7 (−0.9, −0.5)	−	<0.001
Crisps					
Weight (g/week)	67 (66, 69)	3 (0, 5)	5 (1, 8)	−	0.002
Fibre (g/week)	2.7 (2.6, 2.7)	0.1 (0.0, 0.2)	0.2 (0.1, 0.3)	−	0.002
Fibre (% of total fibre)	4.5 (4.4, 4.6)	0.5 (0.3, 0.7)	1.56 (1.3, 1.8)	−	<0.001
Rice/pasta					
Weight (g/week)	268 (262, 273)	−4 (−15, 6)	−46 (−60, −31)	−	<0.001
Fibre (g/week)	1.9 (1.9, 2.0)	0.03 (−0.1, 0.1)	−0.2 (−0.3, −0.1)	−	<0.001
Fibre (% of total fibre)	3.1 (3.1,3.2)	0.2 (0.1, 0.4)	0.2 (0.0, 0.4)	−	0.079
Breakfast cereal					
Weight (g/week)	248 (243, 254)	−19 (−28, −9)	−38 (−51, −25)	−	<0.001
Fibre (g/week)	10.2 (10.0, 10.5)	−0.8 (−1.2, −0.3)	−1.8 (−0.4, −1.2)	−	<0.001
Fibre (% of total fibre)	15.3 (14.9, 15.6)	−0.6 (−1.2, 0.0)	−0.8 (−1.7, 0.0)	−	0.063
Bread					
Weight (g/week)	395 (388, 403)	7 (−7, 21)	9 (−10, 29)	−	0.675
Fibre (g/week)	12.9 (12.4, 13.1)	0.4 (−0.3, 1.0)	0.4 (−0.5, 1.2)	−	1.000
Fibre (% of total fibre)	18.5 (18.1, 18.9)	1.21 (0.5, 2.0)	3.2 (2.2, 4.2)	−	<0.001
Vegetables					
Weight (g/week)	440 (433, 447)	−90 (−102, −78)	−227 (−244, 211)	−	<0.001
Fibre (g/week)	9.9 (9.70, 10.0)	−2.0 (−2.2, −1.7)	−5.0 (−5.3, −4.9)	−	<0.001
Fibre (% of total fibre)	15.7 (15.5, 15.9)	−2.6 (−3.0, −2.3)	−6.8 (−7.3, −6.2)	−	<0.001
Fruits					
Weight (g/week)	384 (378, 389)	−5 (−16, 6)	−56 (−71, −41)	−	<0.001
Fibre (g/week)	5.5 (5.4, 5.6)	−0.1 (−0.2, 0.1)	−0.8 (−1.0, −0.6)	−	<0.001
Fibre (% of total fibre)	8.9 (8.7, 9.0)	0.3 (0.1, 0.6)	0.3 (−0.1, 0.7)	−	0.173
Baked beans					
Weight (g/week)	101 (146, 152)	−12 (−18, −6)	−29 (−37, −21)	−	<0.001
Fibre (g/week)	5.4 (5.3, 5.5)	−0.4 (−0.7, −0.2)	−1.5 (−1.8, −1.2)	−	<0.001
Fibre (% of total fibre)	8.63 (8.5, 8.8)	−0.5 (−1.0, −0.2)	−1.6 (−2.1, −1.1)	−	<0.001
**Combined PE score**^e^					
n	3455	3766	1040	568	
Potatoes					
Chips/roast					
Weight (g/week)	231 (209, 217)	2 (−6, 11)	10 (−3, 12)	5.4 (−11, 22)	1.000
Fibre (g/week)	4.3 (4.2, 4.4)	0.1 (−0.1, 0.3)	0.3 (−0.01, 0.5)	0.2 (−0.1, 0.5)	0.676
Fibre (% of total fibre)	7.1 (7.0, 7.3)	0.6 (0.2, 0.9)	1.5 (1.0, 2.1)	2.2 (1.5, 2.8)	<0.001
Boiled/mashed					
Weight (g/week)	215 (211, 220)	−15 (−24, −7)	−44 (−56, −31)	−95 (−111, −79)	<0.001
Fibre (g/week)	2.5 (2.4, 2.5)	−1.2 (−2.3, −0.1)	−0.5 (−0.7, −0.4)	−1.1 (−1.3, −0.9)	<0.001
Fibre (% of total fibre)	4.0 (3.9, 4.1)	−0.1 (−0.3, 0.1)	−0.3 (−0.6, −0.1)	−1.3 (−1.7, −1.0)	<0.001
Crisps					
Weight (g/week)	67 (66, 67)	2 (−1, 5)	3 (−1, 8)	8 (2, 13)	0.002
Fibre (g/week)	2.7 (2.6, 2.7)	0.1 (−0.02, 0.2)	0.1 (−0.04, 0.3)	0.3 (0.1, 0.5)	0.002
Fibre (% of total fibre)	4.5 (4.4, 4.6)	0.4 (0.2, 0.7)	1.2 (0.8, 1.5)	1.9 (1.5, 2.4)	<0.001
Rice/pasta					
Weight (g/week)	266 (260, 273)	1 (−11, 14)	−31 (−50, −13)	−53 (−77, −30)	<0.001
Fibre (g/week)	1.9 (1.9, 2.0)	0.1 (−0.02, 0.2)	−0.2 (−0.3, −0.02)	−0.2 (−0.4, −0.1)	0.003
Fibre (% of total fibre)	3.1 (3.0, 3.2)	0.3 (0.2, 0.5)	0.2 (−0.02, 0.5)	0.2 (−0.1, 0.5)	0.547
Breakfast cereal					
Weight (g/week)	251 (245, 257)	−22 (−33, −12)	−35 (−51, −18)	−48 (−69, −28)	<0.001
Fibre (g/week)	10.3 (10.1, 10.6)	−0.9 (−1.4, −0.4)	−1.6 (−2.4, −0.8)	−2.4 (−3.3, −1.4)	<0.001
Fibre (% of total fibre)	15.4 (15.0, 15.7)	−0.8 (−1.5, −0.1)	−0.8 (−1.8, 0.3)	−1.7 (−2.7, −0.02)	0.044
Bread					
Weight (g/week)	396 (387, 404)	4 (−13, 20)	1 (−24, 25)	13 (−18, 44)	1.000
Fibre (g/week)	12.9 (12.5, 13.2)	0.3 (−0.5, 1.0)	−0.5 (−1.5, 0.6)	0.8 (−0.6, 2.2)	0.834
Fibre (% of total fibre)	18.5 (18.1, 18.9)	1.2 (0.3, 2.0)	1.7 (0.4, 2.3)	4.6 (2.9, 6.2)	<0.001
Vegetables					
Weight (g/week)	445 (437, 453)	−85 (−99, −71)	−175 (−197, −154)	−256 (−284, −229)	0.001
Fibre (g/week)	10.0 (9.8, 10.1)	−1.9 (−2.2, −1.5)	−3.8 (−4.2, −3.3)	−5.7 (−6.3, −5.1)	<0.001
Fibre (% of total fibre)	15.8 (15.5, 16.0)	−2.4 (−2.8, −1.9)	−4.9 (−5.6, −4.2)	−7.7 (−8.6, −6.8)	<0.001
Fruits					
Weight (g/week)	388 (381, 394)	−6 (−19, 6)	−51 (−70, −32)	−54 (−79, −30)	<0.001
Fibre (g/week)	5.6 (5.5, 5.7)	−0.1 (−0.3, 0.1)	−0.7 (−1.0, −0.5)	−0.8 (−1.1, −0.4)	<0.001
Fibre (% of total fibre)	8.9 (8.8, 9.1)	0.3 (0.0, 0.6)	0.1 (−0.4, 0.6)	0.8 (0.1, 1.4)	0.009
Baked beans
Weight (g/week)	150 (146, 153)	−14 (−21, −7)	−28 (−39, −18)	−54 (−67, −40)	<0.001
Fibre (g/week)	5.4 (5.3, 5.5)	−0.5 (−0.7, −0.3)	−1.0 (−1.4, −0.7)	−1.9 (−2.4, −1.5)	<0.001
Fibre (% of total fibre)	5.7 (8.5, 8.8)	−0.5 (−0.9, −0.1)	−0.9 (−1.5, −0.3)	−2.3 (−3.1, −1.6)	<0.001

FFQ, food frequency questionnaire; PE, picky eating.

a Values are mean (95% CI).

b Values are mean differences (95% CI) from reference category; singletons only.

c P values are for comparison of highest PE score with reference category (0) (ANOVA).

d PE score: Does your child have definite likes and dislikes as far as food is concerned? 0, no; 1, yes, quite choosy; 2, Yes, very choosy.

e Combined PE score: 0, score 0 at both time points (24 months and 38 months); 1, score 1 at either or both time points; 2, score 2 once; 3, score 2 at both time points.

**Table 4:** Picky eating score as a predictor of dietary fibre (non-starch polysaccharide) intake (g/day) from FFQ in children in ALSPAC.

	Diet at 38 months (FFQ)			
	n	R^2^	Unstandardised B (95% CI) (g/day)^a^	P value
PE score at 38 months^b^				
Model 1^c^	9544	0.026	–1.36 (–1.53, –1.19)	<0.001
Model 2^d^	8161	0.054	–1.49 (–1.67, –1.31)	<0.001
Model 3^e^	8204	0.110	–1.40 (–1.58, –1.23)	<0.001
Model 4^f^	8692	0.048	–1.40 (–1.58, –1.22)	<0.001
Model 5^g^	6899	0.125	–1.43 (–1.62, –1.24)	<0.001
Combined PE score^h^				
Model 1^c^	8829	0.028	–1.62 (–1.87, –1.32)	<0.001
Model 2^d^	7686	0.035	–0.98 (–1.19, –0.77)	<0.001
Model 3^e^	7849	0.112	–1.68 (–1.93, –1.44)	<0.001
Model 4^f^	8215	0.049	–1.68 (–1.93, –1.42)	<0.001
Model 5^g^	6666	0.126	–1.70 (–1.96, –1.43)	<0.001

FFQ, food frequency questionnaire; PE, picky eating.

a Coefficients for PE score 2 vs score 0, or combined score 3 vs 0.

b PE score: Does your child have definite likes and dislikes as far as food is concerned? 0, no; 1, yes, quite choosy; 2, yes, very choosy.

c Model 1: minimal adjustment for sex only.

d Model 2: Model 1 + adjusted for maternal education, parity, pre-pregnancy BMI, maternal age, birth weight.

e Model 3: Model 1 + adjusted for maternal diet in pregnancy (fruit and vegetable index: aggregate weight of fruit and vegetable items), Crown–Crisp anxiety sub-scale at 21 months, Edinburgh Postnatal Depression Scale at 21 months.

f Model 4: Model 1 + adjusted for age of introduction of lumpy foods, breast feeding duration.

g Model 5: All models combined.

h Combined PE score: 0, score 0 at both time points (24 months and 38 months); 1, score 1 at either or both time points; 2, score 2 once; 3, score 2 at both time points.

**Table 5:** Association of tertiles of fibre intake from FFQ in children in ALSPAC aged 38 months with stool type at age 42 months from parent-completed questionnaires.

	Dietary fibre intake (g/day, range)	Stool type (hard) at 42 months, odds ratio (95% confidence intervals)		

		Never (ref) (n = 2563)	Sometimes (n = 4516)	Usually (n = 1820)
Dietary fibre intake at 38 months (FFQ)				
Tertile 1 Lowest intake	0.7–<7.4	–	1.29 (1.13, 1.48), p < 0.001	1.87 (1.61, 2.16), p < 0.001
Tertile 2 Medium intake	7.4–<9.7	–	1.33 (1.16, 1.51), p < 0.001	1.47 (1.27, 1.71), p < 0.001
Tertile 3 Highest intake (ref)	9.7–22.5	–	1.00	1.00

FFQ, food frequency questionnaire

**Table 6:** Multinomial modelling of stool type with picky eating score at 38 months in children in ALSPAC: mediation by dietary fibre from an FFQ in an adjusted model.

	n	Odds ratio for stool type (hard) at 42 months (95% confidence intervals)		
		Never (ref)	Sometimes	Usually
Simple relationship: without fibre adjustment^a^				
PE score at 38 months^b^				
0	3235	–	1.00 (ref)	1.00 (ref)
1	2955	–	1.16 (1.01, 1.32), p = 0.031	1.12 (0.97, 1.30), p = 0.137
2	1122	–	1.25 (1.04, 1.51), p = 0.019	1.31 (1.07, 1.61), p = 0.010
Mediated relationship: with fibre adjustment^a^				
PE score at 38 months^b^				
0	3164	–	1.00 (ref)	1.00 (ref)
1	2889	–	1.14 (1.00, 1.30), p = 0.056	1.07 (0.92, 1.24), p = 0.398
2	1088	–	1.18 (0.97, 1.43), p = 0.094	1.16 (0.94, 1.43), p = 0.180

PE, picky eating. Unadjusted results are shown in Supplementary Table 4.

a Adjusted for sex, maternal education, parity, pre-pregnancy BMI, maternal age, birth weight, maternal diet in pregnancy (fruit and vegetable index: aggregate weight of fruit and vegetable items), CrowneCrisp anxiety subscale at 21 months, Edinburgh Postnatal Depression Scale at 21 months, age of introduction of lumpy foods, breast feeding duration (equivalent to model 5 in Table 4), with or without additional adjustment for dietary fibre intake.

b PE score: Does your child have definite likes and dislikes as far as food is concerned? 0, no; 1, yes, quite choosy; 2, yes, very choosy.

## Abbreviations

ALSPAC: Avon Longitudinal Study of Parents and Children
FFQ: food frequency questionnaire
FR: food record
NDNS: National Diet and Nutrition Survey
SACN: Scientific Advisory Committee on Nutrition
