# Supplementary material for: Picky eating in preschool children: Associations with dietary fibre intakes and stool hardness
Source: Appetite. Author manuscript; Available in PMC 2018 Feb 27. (PMC5828098; doi:10.1016/j.appet.2016.02.021)
Supplement: Supplementary data [file NIHMS76244-supplement-Supplementary_data.docx]

**SUPPLEMENTARY TABLE 1**

Fibre and macronutrient intakes from FR in children in ALSPAC 43 months by picky eating score at 38 months and a combined picky eating score for 24 and 38 months

|  | **Picky eating score** | | | |
| --- | --- | --- | --- | --- |
|  | **0^a^** | **1^b^** | **2^b^** | **3** |
| PE score at 38 mo^c^ |  |  |  |  |
| n | 364 | 320 | 131 | - |
| Fibre (g/day) | 8.6 (8.4, 8.9) | -0.7 (-1.2, -0.2)** | -1.5 (-2.2, -0.9)*** | - |
| % below proposed UK RDI^e^ | 82.1 | 88.4 | 93.1 |  |
| Energy (kJ/day) | 5704 (5592, 5817) | -53 (-252, 144) | -73 (-336, 190) | - |
| Carbohydrate (g/day) | 178 (175, 182) | -3 (-10, 3) | -5 (-13, 4) | - |
| Fat (g/day) | 55.2 (53.7, 56.6) | 0.8 (-1.7, 3.2) | 1.7 (-1.7, 5.0) | - |
| Protein (g/day) | 47.9 (46.7, 49.1) | -1.8 (-4.0, 0.3) | -3.2 (-6.1, -0.4)* | - |
| Free sugars (g/day) | 56.7 (54.5, 58.8) | -1.1 (-5.7, 3.5) | 0.9 (-5.2, 7.0) | - |
|  |  |  |  |  |
| Combined PE score^d^ |  |  |  |  |
| n | 300 | 349 | 94 | 50 |
| Fibre (g/day) | 8.7 (8.4, 9.0) | -0.7 (-1.2, -0.2)** | -1.3 (-2.1, -0.4)*** | -2.3 (-3.4, -1.2)*** |
| % below proposed UK RDI^e^ | 81.0 | 88.2 | 90.4 | 98.0 |
| Energy (kJ/day) | 5713 (5590, 5835) | -69 (-290, 152) | -50 (-383, 282) | -31 (-460, 398) |
| Carbohydrate (g/day) | 180 (176, 183) | -5 (-12, 2) | -5 (-16, 6) | -7 (-21, 8) |
| Fat (g/day) | 54.9 (53.3, 56.5) | 1.1 (-1.7, 3.9) | 1.9 (-2.3, 6.1) | 3.4 (-2.0, 8.9) |
| Protein (g/day) | 48.0 (46.6, 49.3) | -1.8 (-4.2, 0.6) | -2.6 (-6.3, 1.0) | -2.8 (-7.5, 1.9) |
| Free sugars (g/day) | 57.3 (54.9, 59.6) | -2.3 (-6.7, 2.1) | 0.7 (-6.0, 7.3) | 0.9 (-7.7, 9.5) |

FR food record; PE, picky eating.

Dietary fibre is measured as non-starch polysaccharide (NSP).

^a^Values are mean (95% CI); singletons only.

^b^Values are mean differences (95% CI) from reference category; singletons only.

^c^PE score: Does your child have definite likes and dislikes as far as food is concerned? 0, no; 1, yes, quite choosy; 2, Yes, very choosy.

^d^Combined PE score: 0, score 0 at both time points (24 months and 38 months); 1, score 1 at either or both time points; 2, score 2 once; 3, score 2 at both time points.

^e^Proposed UK guideline of 15 g AOAC fibre/day for children ages 2–5 years old (equivalent to 11 g NSP fibre/day) (Scientific Advisory Committee on Nutrition, 2014). {Scientific Advisory Committee on Nutrition, 2014 #247}

Values significantly different from 0 category: *p≤0.05,**p≤0.01,***p≤0.001 (ANOVA with multiple comparisons).

**SUPPLEMENTARY TABLE 2**

Main food group sources of dietary fibre (non-starch polysaccharide) by picky eating score (food weight in g/week, g fibre/week and % of total fibre) assessed in a subsample of children in ALSPAC by parental-completion FR at age 43 months

|  | **Diet at 43 months (FR)** | | | | |
| --- | --- | --- | --- | --- | --- |
|  | **0^a^** | **1^b^** | **2^b^** | **3** | **P value^c^** |
| **PE score at 38 mo^d^** |  |  | |  |  |
| n | 364 | 320 | 131 | - |  |
| Potatoes |  |  |  |  |  |
| Chips/roast |  |  |  |  |  |
| Weight (g/week) | 232 (206, 257) | -16 (-60, 28) | 4 (-54, 63) | - | 0.584 |
| Fibre (g/week) | 4.7 (4.2, 5.3) | -0.3 (-0.6, 1.2) | 0.2 (-1.0, 1.4) | - | 0.585 |
| Fibre (% of total fibre) | 8.1 (7.2, 9.0) | 0.3 (-1.4, 1.9) | 2.2 (-0.01, 4.4) | - | 0.053 |
| Boiled/mashed |  |  |  |  |  |
| Weight (g/ week) | 184 (162, 206) | -7 (-46. 32) | -35 (-86, 17) | - | 0.269 |
| Fibre (g/week) | 2.4 (2.1, 2.7) | -0.1 (-0.7, 0.4) | -0.6 (-1.3, 0.1) | - | 0.113 |
| Fibre (% of total fibre) | 3.9 (3.5, 4.4) | 0.3 (-0.6, 1.2) | -0.2 (-1.5, 1.0) | - | 0.576 |
| Crisps |  |  |  |  |  |
| Weight (g/ week) | 77 (68, 85) | 0 (-14, 14) | 5 (-14, 23) | - | 0.793 |
| Fibre (g/week) | 3.2 (2.8, 3.6) | -0.3 (-0.9, 0,4) | 0.1 (-0.8, 0.9) | - | 0.538 |
| Fibre (% of total fibre) | 5.6 (4.9, 6.3) | 0.3 (-0.9, 1.6) | 1.6 (-0.04, 3.3) | - | 0.064 |
| Rice/pasta |  |  |  |  |  |
| Weight (g/week) | 245 (215, 275) | 41 (-17, 98) | -14 (-90, 62) | - | 0.127 |
| Fibre (g/week) | 2.1 (1.9, 2.4) | 0.3 (-0.2, 0.9) | -0.2 (-0.9, 0.5) | - | 0.153 |
| Fibre (% of total fibre) | 3.8 (3.3, 4.4) | 0.9 (-0.2, 1.9) | 0.5 (-0.9, 1.9) | - | 0.129 |
| Breakfast cereal |  |  |  |  |  |
| Weight (g/week) | 170 (155, 185) | -10 (-36, 16) | -27 (-62, 7) | - | 0.163 |
| Fibre (g/week) | 7.8 (7.0, 8.6) | -1.1 (-2.6, 0.4) | -1.3 (3.2, 0.7) | - | 0.121 |
| Fibre (% of total fibre) | 12.3 (11.1, 13.5) | -0.9 (-3.0, 1.3) | -0.1 (-3.0, 2.7) | - | 0.598 |
| Bread |  |  |  |  |  |
| Weight (g/week) | 367 (347, 388) | 10 (-27, 47) | -29 (-79, 20) | - | 0.162 |
| Fibre (g/week) | 10.1 (9.3, 10.8) | -0.1 (-1.5, 1.4) | -1.6 (3.5, 0.4) | - | 0.129 |
| Fibre (% of total fibre) | 16.4 (15.4, 17.5) | 1.0 (-1.1, 3.1) | 0.8 (-2.0, 3.6) | - | 0.518 |
| Vegetables |  |  |  |  |  |
| Weight (g/week) | 325 (297, 354) | -54 (-101, -8) | -156 (-218, -95) | - | <0.001 |
| Fibre (g/week) | 7.6 (7.0, 8.3) | -1.3 (-2.4, -0.2) | -3.7 (-5.2, -2.3) | - | <0.001 |
| Fibre (% of total fibre) | 12.6 (11.6, 13.6) | -1.5 (-3.3, 0.3) | -4.8 (-7.2, -2.5) | - | <0.001 |
| Fruits |  |  |  |  |  |
| Weight (g/week) | 522 (476, 569) | -30 (-111, 50) | -173 (-280, -66) | - | <0.001 |
| Fibre (g/week) | 7.4 (6.7, 8.1) | -0.3 (-1.5, 1.0) | -2.4 (-4.0, -0.7) | - | 0.002 |
| Fibre (% of total fibre) | 11.8 (10.9, 12.8) | 0.5 (-1.3, 2.4) | -2.2 (-4.6, 0.3) | - | 0.032 |
| Baked beans |  |  |  |  |  |
| Weight (g/week) | 123 (103, 143) | -30 (-63, 4) | -13 (-57, 32) | - | 0.112 |
| Fibre (g/week) | 4.4 (3.7, 5.1) | -1.1 (-2.3, 0.1) | -0.4 (-2.0, 1.2) | - | 0.100 |
| Fibre (% of total fibre) | 6.9 (5.8, 8.0) | -1.3 (-3.2, 0.6) | -0.1 (-2.6, 2.4) | - | 0.212 |
|  |  |  |  |  |  |
| **Combined PE score^e^** |  |  |  |  |  |
| n | 300 | 349 | 94 | 50 |  |
| Potatoes |  |  |  |  |  |
| Chips/roast |  |  |  |  |  |
| Weight (g/week) | 241 (212, 270) | -32 (-82, 18) | -22 (-97, 53) | -2 (-99, 95) | 0.376 |
| Fibre (g/week) | 4.9 (4.3, 5.5) | -0.6 (-1.7, 0.4) | 0.4 (-2.0, 1.1) | 0.1 (-1.9, 2.1) | 0.374 |
| Fibre (% of total fibre) | 8.4 (7.4, 9.4) | -0.4 (-2.2, 1.5) | 0.6 (-2.2, 3.5) | 3.1 (-0.5, 6.8) | 0.081 |
| Boiled/mashed |  |  |  |  |  |
| Weight (g/week) | 187 (164, 211) | -17(-61, 27) | -11 (-76, 55) | -47 (-132, 37) | 0.454 |
| Fibre (g/week) | 2.5 (2.1, 2.8) | -0.3 (-0.9, 0.4) | -0.3 (-1.2, 0.6) | -0.8 (-2.0, 0.4) | 0.318 |
| Fibre (% of total fibre) | 4.0 (3.5, 4.5) | 0.1 (-1.0, 1.1) | 0.4 (-1.2, 2.0) | -0.4 (-2.5, 1.7) | 0.806 |
| Crisps |  |  |  |  |  |
| Weight (g/week) | 77 (68, 87) | 1 (-17, 15) | 3 (-21, 26) | 1 (-30, 31) | 0.982 |
| Fibre (g/week) | 3.2 (2.8, 3.7) | -0.2 (-0.9, 0.5) | -0.2 (-1.3, 0.9) | 0.1 (-1.3, 1.5) | 0.843 |
| Fibre (% of total fibre) | 5.6 (4.8, 6.3) | 0.4 (-1.0, 1.9) | 0.3 (-1.8, 2.5) | 2.8 (0.1, 5.6) | 0.064 |
| Rice/pasta |  |  |  |  |  |
| Weight (g/week) | 239 (207, 270) | 46 (-19, 111) | 31 (-66, 129) | -55 (-181, 70) | 0.080 |
| Fibre (g/week) | 2.1 (1.8, 2.4) | 0.4 (-0.2, 1.0) | 0.1 (-0.9, 0.9) | -0.5 (-1.6, 0.7) | 0.112 |
| Fibre (% of total fibre) | 3.8 (3.2, 4.4) | 0.9 (-0.3, 2.1) | 0.6 (-1.1, 2.4) | 0.2 (-2.0, 2.5) | 0.227 |
| Breakfast cereal |  |  |  |  |  |
| Weight (g/week) | 170 (153, 187) | -5 (-35, 25) | -31 (-76, -14) | -25 (-83, 33) | 0.247 |
| Fibre (g/week) | 7.9 (6.9, 8.8) | -1.0 (-2.6, 0.7) | -0.9 (-3.4, 1.6) | -2.1 (-5.4, 1.1) | 0.228 |
| Fibre (% of total fibre) | 12.2 (10.9, 13.5) | -0.6 (-3.0, 1.9) | 0.7 (-3.0, 4.4) | -0.5 (-5.3, 4.2) | 0.788 |
| Bread |  |  |  |  |  |
| Weight (g/week) | 366 (344, 387) | 12 (-30, 53) | -42 (-104, 20) | -2 (-82, 78) | 0.142 |
| Fibre (g/week) | 10.0 (9.2, 10.9) | 0.2 (-1.5, 1.8) | -2.6 (-5.1, -0.2) | -0.1 (-3.3, 3.0) | 0.019 |
| Fibre (% of total fibre) | 16.3 (15.1, 17.5) | 1.3 (-1.0, 3.7) | -2.6 (-6.1, 1.0) | 6.4 (1.8, 11.0) | <0.001 |
| Vegetables |  |  |  |  |  |
| Weight (g/week) | 274 (249, 299) | -50 (-102, 3) | -127 (-205, -49) | -176 (-277, -75) | <0.001 |
| Fibre (g/week) | 7.6 (6.9, 8.3) | -1.2 (-2.4, -0.01) | -3.0 (-5.7, -1.0) | -4.6 (-6.9, -2.2) | <0.001 |
| Fibre (% of total fibre) | 12.4 (11.3, 13.5) | -1.2 (-3.2, 0.8) | -3.4 (-6.3, -0.4) | -6.3 (-10.1, -2.5) | <0.001 |
| Fruits |  |  |  |  |  |
| Weight (g/week) | 534 (482, 588) | -36 (-128, 55) | -114 (-250, 23) | -276 (-453, -100) | <0.001 |
| Fibre (g/week) | 7.6 (6.8, 8.4) | -0.5 (-1.9, 0.9) | -1.6 (-3.7, 0.5) | -4.0 (-6.7, -1.3) | 0.001 |
| Fibre (% of total fibre) | 12.0 (11.0, 13.1) | 0.3 (-1.8, 2.3) | -0.6 (-3.7, 2.5) | -4.6 (-8.6, -0.6) | 0.014 |
| Baked beans |  |  |  |  |  |
| Weight (g/week) | 127 (105, 150) | -36 (-74, 2) | 18 (-39, 75) | -80 (-153, -6) | 0.002 |
| Fibre (g/week) | 4.6 (3.8, 5.3) | -1.3 (-2.6, 0.1) | 0.7 (-1.3, 2.8) | -2.8 (-5.4, -0.2) | 0.001 |
| Fibre (% of total fibre) | 7.1 (5.9, 8.4) | -1.7 (-3.8, 0.4) | 1.8 (-1.4, 4.9) | -4.2 (-8.2, -0.1) | 0.001 |

FR, food record; PE, picky eating.

^a^Values are mean (95% CI).

^b^Values are mean differences (95% CI) from reference category; singletons only.

^c^P values are for comparison of highest PE score with reference category (0) (ANOVA).

^d^PE score: Does your child have definite likes and dislikes as far as food is concerned? 0, no; 1, yes, quite choosy; 2, Yes, very choosy.

^e^Combined PE score: 0, score 0 at both time points (24 months and 38 months); 1, score 1 at either or both time points; 2, score 2 once; 3, score 2 at both time points.

**SUPPLEMENTARY TABLE 3**

Picky eating score as a predictor of fibre (non-starch polysaccharide) intake (g/day) from FR in a subsample of children in ALSPAC

|  | **n** | **Diet at 43 months (FR)** | | |
| --- | --- | --- | --- | --- |
|  |  | **R^2^** | **Unstandardised B (95% CI) (g/day)** | **P value** |
| PE score at 38 months^a^ |  |  |  |  |
| Model 1^b^ | 815 | 0.058 | -1.55 (-2.09, -1.01) | <0.001 |
| Model 2^c^ | 731 | 0.083 | -1.55 (-2.11, -1.00) | <0.001 |
| Model 3^d^ | 738 | 0.117 | -1.55 (-2.10, -1.003) | <0.001 |
| Model 4^e^ | 764 | 0.070 | -1.61 (-2.16, -1.07) | <0.001 |
| Model 5^f^ | 645 | 0.126 | -1.37 (-1.95, -0.79) | <0.001 |
| Combined PE score^g^ |  |  |  |  |
| Model 1^b^ | 793 | 0.063 | -2.32 (-3.12, -1.52) | <0.001 |
| Model 2^c^ | 716 | 0.092 | -2.19 (-3.02, -1.36) | <0.001 |
| Model 3^d^ | 729 | 0.126 | -2.14 (-2.93, -1.34) | <0.001 |
| Model 4^e^ | 755 | 0.080 | -2.29 (-3.09, -1.48) | <0.001 |
| Model 5^f^ | 641 | 0.131 | -1.88 (-2.73, -1.04) | <0.001 |

FR, food record; PE, picky eating.

Coefficients for PE score 2 vs score 0, or combined score 3 vs 0.

^a^PE score: Does your child have definite likes and dislikes as far as food is concerned? 0, no; 1, yes, quite choosy; 2, Yes, very choosy.

^b^Model 1: minimal adjustment for sex only.

^c^Model 2: Model 1 + adjusted for maternal education, parity, pre-pregnancy BMI, maternal age, birth weight.

^d^Model 3: Model 1 + adjusted for maternal diet in pregnancy (fruit and vegetable index: aggregate weight of fruit and vegetable items), Crown–Crisp anxiety subscale at 21 months, Edinburgh Postnatal Depression Scale at 21 months.

^e^Model 4: Model 1 + adjusted for age of introduction of lumpy foods, breast feeding duration.

^f^Model 5: All models combined.

^g^Combined PE score: 0, score 0 at both time points (24 months and 38 months); 1, score 1 at either or both time points; 2, score 2 once; 3, score 2 at both time points.

**SUPPLEMENTARY TABLE 4**

Association of stool type at 42 months with picky eating score at 38 months and picky eating score at 24 and 38 months combined in children in ALSPAC

|  | **Stool type (hard) at 42 months** | | | **P value (chi square)** |
| --- | --- | --- | --- | --- |
|  | **Never** | **Sometimes** | **Usually** |  |
| Picky eating score at 38 months^a^ |  |  |  |  |
| 0 | 907 (22.2%) | 2006 (49.1%) | 1176 (28.8%) | 0.002 |
| 1 | 727 (19.7%) | 1911 (51.8%) | 1049 (28.5%) |  |
| 2 | 242 (17.9%) | 694 (51.3%) | 418 (30.9%) |  |
| Combined picky eating score^b^ |  |  |  |  |
| 0 | 739 (22.2%) | 1640 (49.3%) | 948 (28.5%) | 0.006 |
| 1 | 756 (20.6%) | 1890 (51.5%) | 1027 (28.0%) |  |
| 2 | 186 (18.4%) | 503 (49.7%) | 324 (32.0%) |  |
| 3 | 94 (16.8%) | 299 (53.5%) | 166 (29.7%) |  |

Values are n (%).

^a^Picky eating score: Does your child have definite likes and dislikes as far as food is concerned? 0, no; 1, yes, quite choosy; 2, Yes, very choosy.

^b^Combined PE score: 0, score 0 at both time points (24 months and 38 months); 1, score 1 at either or both time points; 2, score 2 once; 3, score 2 at both time points.

**SUPPLEMENTARY TABLE 5**

Multinomial modelling of stool type with picky eating score in children in ALSPAC: mediation by fibre intake from FFQ in unadjusted model

|  | **n** | **OR for stool type (hard) at 42 months (95% CI)** | | |
| --- | --- | --- | --- | --- |
|  |  | **Never (ref)** | **Sometimes** | **Usually** |
| Simple relationship: without fibre adjustment | | | | |
| PE score at 38 months^a^ |  |  |  |  |
| 0 | 4089 | - | 1.00 (ref) | 1.00 (ref) |
| 1 | 3687 | - | 1.19 (1.10, 1.34), p=0.004 | 1.11 (0.98, 1.27), p=0.102 |
| 2 | 1354 | - | 1.30 (1.06, 1.34), p=0.002 | 1.33 (1.11, 1.60), p=0.002 |
| Mediated relationship: with fibre adjustment | | | | |
| PE score at 38 months^a^ |  |  |  |  |
| 0 | 3971 | - | 1.00 (ref) | 1.00 (ref) |
| 1 | 3589 | - | 1.16 (1.03, 1.30), p=0.015 | 1.04 (0.92, 1.19), p=0.532 |
| 2 | 1312 | - | 1.20 (1.10, 1.43), p=0.034 | 1.14 (0.94, 1.37), p=0.174 |

PE, picky eating.

^a^PE score: Does your child have definite likes and dislikes as far as food is concerned? 0, no; 1, yes, quite choosy; 2, Yes, very choosy.

**SUPPLEMENTARY FIGURE 1**

Participant flowchart

Complete FFQ at 38 months

n=8829

Pregnant women enrolled into ALSPAC

n=14,541

Live births

n=14,062

Alive at 1 year

n=13,988

Picky eating question answered
at 38 months

n=9844

Picky eating question answered
at 24 and 38 months

n=9080

Did not answer:

1. n=3479

2. n=3769

3. n=4533

4. n=300

5. n=251

6. n=714

7. n=508

Complete FFQ at 38 months

n=9544

Question on stool hardness completed at 42 months

n=8872

Question on stool hardness completed at 42 months

n=8350

Picky eating question answered at 24 months

n=10,134

1

2

3

4

5

6

7

Excluded: multiple births

n = 375

Eligible participants

n = 13,613
